# Association between *IRF6, TP63, GREM1* Gene Polymorphisms and Non-Syndromic Orofacial Cleft Phenotypes in Vietnamese Population: A Case–Control and Family-Based Study

**DOI:** 10.3390/genes14111995

**Published:** 2023-10-25

**Authors:** Loc Nguyen Gia Pham, Teruyuki Niimi, Satoshi Suzuki, Minh Duc Nguyen, Linh Cao Hoai Nguyen, Tuan Duc Nguyen, Kien Ai Hoang, Duc Minh Nguyen, Chisato Sakuma, Toko Hayakawa, Makino Hiyori, Nagana Natsume, Hiroo Furukawa, Hideto Imura, Junko Akashi, Tohru Ohta, Nagato Natsume

**Affiliations:** 1 Division of Research and Treatment for Oral and Maxillofacial Congenital Anomalies, Aichi Gakuin University, 2–11 Suemori-dori, Chikusa-ku, Nagoya 464-8651, Japan; gialocp@gmail.com (L.N.G.P.); niimi@dpc.agu.ac.jp (T.N.); zsatoshi@hotmail.com (S.S.); minhduc.dentist@gmail.com (D.M.N.); char_nyoko@yahoo.co.jp (C.S.); ag223d15@dpc.agu.ac.jp (N.N.); h-imura@dpc.agu.ac.jp (H.I.);; 2 Odonto-Maxillo Facial Hospital of Ho Chi Minh City, 263-265 Tran Hung Dao Street, District 1, Ho Chi Minh City 71000, Vietnam; dr.ducminhrhm@gmail.com (M.D.N.); hoailinh.nguyen.qn@gmail.com (L.C.H.N.); tuan.nd1985@gmail.com (T.D.N.); hoangaikien@gmail.com (K.A.H.); 3 Cleft Lip and Palate Center, Aichi Gakuin Dental Hospital, 2-11 Suemori-dori, Chikusa-ku, Nagoya 464-8651, Japan; hfuru@dpc.agu.ac.jp; 4 Division of Speech, Hearing, and Language, Aichi Gakuin Dental Hospital, 2-11 Suemori-dori, Chikusa-ku, Nagoya 464-8651, Japan; hayakawa@dpc.agu.ac.jp (T.H.); hiyori@dpc.agu.ac.jp (M.H.); 5 School of Odonto-Stomatology, Hanoi Medical University, Hanoi 10000, Vietnam; 6 Advanced Research Promotion Center, Health Sciences University of Hokkaido, Ishikari-Tobetsu 061-0293, Japan; ohta@hoku-iryo-u.ac.jp

**Keywords:** *IRF6*, *TP63*, *GREM1*, cleft lip and palate, cleft lip with or without cleft palate

## Abstract

This study aims to identify potential variants in the *TP63–IRF6* pathway and *GREM1* for the etiology of non-syndromic orofacial cleft (NSOFC) among the Vietnamese population. By collecting 527 case–parent trios and 527 control samples, we conducted a stratified analysis based on different NSOFC phenotypes, using allelic, dominant, recessive and over-dominant models for case–control analyses, and family-based association tests for case–parent trios. Haplotype and linkage disequilibrium analyses were also conducted. *IRF6* rs2235375 showed a significant association with an increased risk for non-syndromic cleft lip and palate (NSCLP) and cleft lip with or without cleft palate (NSCL/P) in the G allele, with p_allele_ values of 0.0018 and 0.0003, respectively. Due to the recessive model (*p* = 0.0011) for the NSCL/P group, the reduced frequency of the GG genotype of rs2235375 was associated with a protective effect against NSCL/P. Additionally, offspring who inherited the G allele at rs2235375 had a 1.34-fold increased risk of NSCL/P compared to the C allele holders. *IRF6* rs846810 and a G-G haplotype at rs2235375–rs846810 of *IRF6* impacted NSCL/P, with *p*-values of 0.0015 and 0.0003, respectively. In conclusion, our study provided additional evidence for the association of *IRF6* rs2235375 with NSCLP and NSCL/P. We also identified *IRF6* rs846810 as a novel marker associated with NSCL/P, and haplotypes G-G and C-A at rs2235375–rs846810 of *IRF6* associated with NSOFC.

## 1. Introduction

To date, approximately 1 in 700 children worldwide [1], and 1.4 out of 1000 live births in Vietnam [2], are born with orofacial clefts (OFCs), and many of them experience a range of functional, psychological and aesthetic problems, including feeding, hearing and speech difficulties [1]. Depending on the region, ethnicity and socioeconomic status, the prevalence of OFC differs and it is observed more frequently among males than females. The majority of OFCs are non-syndromic orofacial cleft (NSOFC), while approximately 30–50% are syndromic [3]. The most popular classification divides NSOFC into four major forms, including cleft lip and palate (NSCLP), cleft lip only (NSCLO), cleft palate only (NSCPO) and atypical facial cleft. Besides this, NSCLP and NSCLO are also combined in a unique group called cleft lip with or without cleft palate (NSCL/P) due to the resemblance of both the epidemiologic features and embryologic timing [4]. The identification of the etiology of birth defects, including NSOFC, has been explored for centuries and the possible contribution of genetic and environmental risk factors has been a subject of debate. In essence, genes, endogenous and exogenous environmental factors, and the interplay between multiple genes and the environment, are all widely accepted to play a role through different mechanisms [4].

Recently, there has been significant statistical and biological support for several susceptibility loci of NSOFC. Through genome-wide association studies (GWAS), researchers have successfully identified common and rare variants, and their interactions with environmental factors [5,6,7]. Of these loci, 1q32, which includes Interferon Regulatory Factor 6 (*IRF6*) that belongs to a family of transcription factors, plays a crucial role in the balance between immature ectoderm differentiation and proliferation during craniofacial morphogenesis. Kondo et al. [8] cloned human *IRF6*, which is well-documented as a causal gene for Van der Woude syndrome (VWS) and Popliteal Pterygium Syndrome, two disorders that can include isolated cleft lip (CLO) and isolated cleft palate (CPO) anomalies. Variants in recognized genes now explain approximately 20% of the pathogenesis of NSOFC, of which variants in *IRF6* account for 12% [9]. In the last few decades, GWAS have identified at least 43 genes/loci associated with NSOFC, and *IRF6* is one of the most related genes among different populations [5,10].

The linkage between p63 and *IRF6* was established with *IRF6* being the direct target of *TP63*, which is located on chromosome 3q28 and encodes the tumor protein p63, during palatal development [11]. The p63 protein, another transcription factor, plays an essential role in regulating ectodermal cell differentiation and embryonic development, including the development of the epidermis, limbs and craniofacial tissues [12]. It has been proven that p63 has a role in activating *IRF6* transcription via the *IRF6* enhancer element called MCS9.7 [13]; a mutation within this enhancer element increases the chances of NSCL/P [14]. Some studies recently reported that PBX proteins, transcription factors in a homeobox protein family, and SHH, Sonic Hedgehog signaling protein, are essential for embryonic development and bind to midfacial regulatory elements to regulate the expression of wingless-type MMTV integration site (WNT) signaling at the lambdoidal junction (where the maxillary, medial nasal and lateral nasal processes fuse) [15,16]. WNT signaling is effective in most tissues during craniofacial development and required for body axis patterning, cell fate specification, cell proliferation [17,18] and palatogenesis [19]. In turn, WNT signaling of *Wnt9b-Wnt3* controls p63, which belongs to a feedback loop with *Irf6*, the mouse ortholog of *IRF6* [15,16]. Another factor that affects *IRF6* through binding to the MCS9.7 enhancer on the binding site in the *IRF6* is the AP2A transcription factor, whose gene *TFAP2A* was reported to be responsible for the cleft etiology in our previous study [20].

Gremlin 1 (*GREM1*) was initially described as an association factor of NSOFC risk due to displaying suggestive genome-wide significance in GWAS [5]. *GREM1* encoded a protein in the bone morphogenetic protein (BMP) antagonist family and mediated the regulation of the dynamic interactions of various epithelial and mesenchymal signaling centers. GREM1 binds to BMP2, BMP4 and BMP7, all of which are expressed in the developing palatal shelves and prevent engagement [21]. GREM1 can bind to a BMP4 precursor intracellularly, preventing BMP4 secretion and, consequently, creating GREM1–BMP4 interactions. GREM1 is also essential for early limb outgrowth and patterning because of its role in the SHH–GREM1–FGF feedback loop [22]. FGF, fibroblast growth factor, plays a role in cellular proliferation, migration and differentiation, mitogenesis, angiogenesis, embryogenesis and wound healing [23]. The FGF signaling pathway regulates multiple developmental processes, including palatogenesis [24].

While several pieces of evidence support the contribution of *IRF6* and its related genes to the etiology of NSOFC, deeper insights into the various subtypes of NSOFC should be obtained. In the present study, to assess whether a statistical association exists between the disease trait and the selected markers, we evaluated the hypothesis that genetic variants located in and around the *IRF6*, *TP63* and *GREM1* genes are associated with pathological NSOFC phenotypes in the Vietnamese population by using case–parent trios and case–control analysis.

## 2. Materials and Methods

### 2.1. Sample Study

This study was based on combining 2 complementary approaches: case–parent trio (a case and its parents) study and case–control study. All the affected participants were carefully screened for the absence of associated anomalies or syndromes by the maxillofacial surgeon and diagnosed with NSOFC based on clinical examination, treatment and medical records at the Odonto and Maxillofacial Hospital of Ho Chi Minh City (HCMC) in Vietnam from 2013 to 2019. A total of 527 Vietnamese patient–unaffected parent trios were included in this study. Of these, 101 trios had an index case with NSCPO, 172 trios with NSCLO and 254 trios with NSCLP. Besides these, 527 ethnically and region-matched healthy controls without a cleft history in their families were recruited for a case–control design. All subjects self-identified as Vietnamese, providing their name, gender and age and identifying as the Kinh people. All cases were selected strictly according several inclusion criteria, including (1) congenital NSOFC presence; (2) no other congenital malformations or acquired diseases of other systemic organs; (3) no family history of other genetic diseases; (4) both parents of the patient were mentally healthy and willing to complete the questionnaire. Inclusion criteria for the unrelated control group were as follows: (1) the absence of any congenital diseases or malformations of the systemic organs; (2) no family history of NSOFC or other genetic diseases for at least three generations; and (3) sound mental health and a willingness to participate in the questionnaire. People who did not meet the inclusion criteria were ruled out from the study, both in case and control samples.

The estimated sample size, power and effect size calculation were calculated by requirement in the case of a 1:1 ratio to achieve 80% statistical power under the assumption of a 5% α level for the case–control and case–parent designs.

Peripheral blood samples were collected on dried blood cards and stored at the World Cleft Gene Bank in Aichi Gakuin University, Nagoya, Japan. In both the case and control samples, informed consent for participation was collected from all participants, either from the participants themselves or from their parents in the case of children under the age of 18. All procedures were performed in accordance with the ethical guidelines laid down by the Declaration of Helsinki (World Medical Association 2013), and the study was approved by the Aichi Gakuin University Ethics Committee (Number 78).

### 2.2. Single-Nucleotide Polymorphism Selection and Genotyping

Single-nucleotide polymorphism (SNP) markers of interest were searched against the dbSNP database (http://www.ncbi.nlm.nih.gov/projects/SNP/ (accessed on 13 January 2022)) and the 1000 Genomes Browser (http://browser.1000genomes.org/index.html (accessed on 13 January 2022)) to determine whether they had previously been reported and whether they had been described as pathogenic or nonpathogenic. In total, 5 SNPs (2 for *IRF6*: rs2235375, rs846810; 2 for *GREM1*: rs2280738, rs1258763; and 1 for *TP63*: rs9332461) were selected for genotyping. The common criterion for selecting the SNP was a minor allele frequency (MAF) >5% from the 1000 Genomes database of the Asian population [25]. Individually, rs2235375 is located in intron 6 of *IRF6* and has been discovered to have a strong correlation with rs2235371 in exon 7, which alters the conserved amino acid valine to isoleucine at codon 274 (p.V274I) in the SMIR-binding domain of *IRF6* [26]. Rs2235371 (the p.V274I polymorphism) is the most significant SNP of *IRF6* associated with NSOFC and has been reported to have statistical associations in various populations [9]. In addition, rs9332461 of *TP63* and rs2280738 and rs1258763 of *GREM1* were selected for genotyping based on previous GWAS and association studies [5,27]. Besides these known associated SNPs, we chose rs846810 of *IRF6*, which has not previously been announced as a causative variant of NSOFC in GWAS and replication studies. *IRF6* rs846810 is located in intron 5 of *IRF6* and is considered a potential marker for the linkage disequilibrium (LD) test in *IRF6*. Furthermore, rs846810 plays roles in transcriptional regulation by changing the binding of transcriptional factors generated by HaploReg v4.2 (https://pubs.broadinstitute.org/mammals/haploreg/haploreg.php (accessed on 6 July 2023)).

The QIAamp DNA Blood Mini Kit (QIAGEN) was used to extract genomic DNA from dried blood spots according to the manufacturer’s instructions. The purity of the DNA was confirmed by spectrophotometric tests. All samples were successfully genotyped, with a genotype call rate of >98%, using the TaqMan^TM^ SNP Genotyping Assay (Applied Biosystems, Foster City, CA, USA) in the 7900 HT Fast Real-Time PCR system (Applied Biosystems, Foster City, CA, USA), according to the manufacturer’s guidelines.

### 2.3. Statistical Analysis

We tested each SNP for an association with NSOFC subtypes. Adherence to Hardy–Weinberg equilibrium (HWE) was assessed for all SNPs using the healthy control group. In the case–control study, the allelic (Allele), genotypic (Geno), dominant (Dom), recessive (Rec) and over-dominant (Over) models of each marker among samples were compared with Chi-square tests and 95% confidence intervals (CIs) for the odds ratios (ORs). In the dominant model, the major allele homozygote effect was compared with the combining effect of heterozygotes and minor allele homozygotes. In the recessive model, the combining effect of the major allele homozygotes and heterozygotes was compared with minor allele homozygotes. The over-dominant model showed a difference in the heterozygote effect compared with the combining effect of the major and minor allele homozygotes. We also implemented the transmission disequilibrium test (TDT), the parent-of-origin effects (POO) and SNP × SNP epistasis for a case–control population-based sample analysis in PLINK (v1.07; http://pngu.mgh.harvard.edu/purcell/plink/ (accessed on 16 March 2023)), which generated an asymptotic *p*-value. The LD and haplotype with each phenotype in the case–control and case–parent analysis were computed by using Haploview 4.2 (http://www.broad.mit.edu/mpg/haploview/ (accessed on 16 March 2023)). Haplotypes with a frequency < 0.03 in these cleft trios were considered rare and thus excluded from statistical analysis.

Bonferroni corrections for multiple SNPs were applied. We used Bonferroni’s correction for 25 tests (5 SNPs × 5 phenotypic groups) and 30 tests (6 haplotypes × 5 phenotypic groups) to determine the critical thresholds in formal significance for the allelic association study, SNP interactions, TDT and POO tests (*p* = 0.002) and haplotype analysis (*p* = 0.0017), respectively.

## 3. Results

### 3.1. Baseline Characteristics

Table 1 displays the characteristics of the study sample. In total, the study included 527 children with NSOFC. In all NSOFC cases, NSCPO (19.2%) was rarer than NSCL/P (80.8%). Among NSCL/P patients, unilateral cleft lip with or without cleft palate (NSUCL/P) (76.3%) was more common than bilateral cleft lip with or without cleft palate (NSBCL/P) (23.7%), with the right-side NSUCL/P (34.8%) being less common than the left-side NSUCL/P (65.2%). A remarkable gender difference was seen in the NSBCL/P group (*p* = 0.01), with more male patients (70.3%) than female patients (29.7%).

### 3.2. Single-Marker Association Analysis

The results of the HWE test and MAF are listed in Table 2, with none of the tested polymorphisms showing a significant deviation from HWE in healthy individuals (*p* > 0.05). The MAF for analyzed nucleotide variants was at least 6%, similar to the MAFs reported in the KHV population (Kinh in Ho Chi Minh City, Vietnam) from the 1000 Genomes database [25].

### 3.3. Case–Control Comparison and Haplotype Analysis

In the *IRF6* gene, rs2235375 showed a significant association with NSCLP (*p* = 0.0018) and NSCL/P (*p* = 0.0003) in the allelic model. A low *p*-value in the genotypic model (p_Geno_) was found for rs2235375 in NSCL/P (*p* = 0.0014), which appeared within a significance level of *p* ≤ 0.002 after the Bonferroni correction (Table 3). Under the assumption of a recessive inheritance, the calculated *p*-value of the recessive genetic model (p_Rec_) for rs2235375 in NSCL/P was 0.0011. *IRF6* rs846810 showed a significant difference in allele frequency between NSCL/P and the control group, with a *p*-value of 0.0015 and OR of 1.41 with 95% CI 1.14–1.74. None of these SNPs showed a significant association with NSCPO or NSCLO (Table 3).

Regarding the *TP63* gene, we did not detect a significant association between NSOFC phenotypes and rs9332461 within the *TP63* promoter region (Table 3).

**Figure 1:**
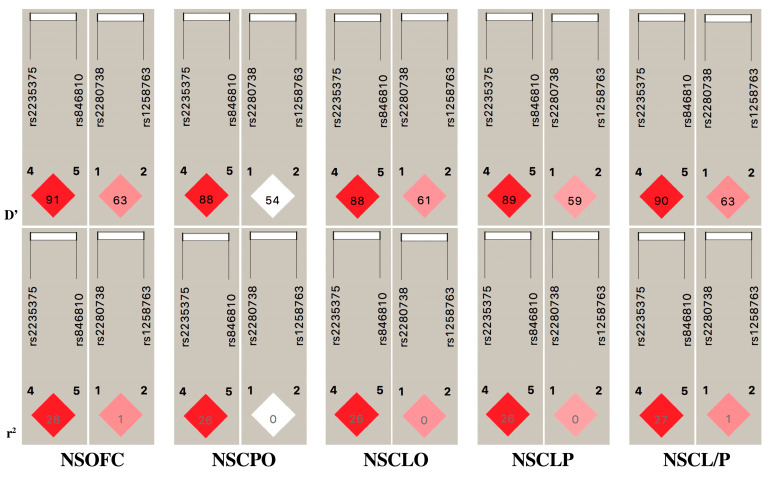
Linkage disequilibrium (LD) analysis in each subtype of NSOFC; the values within each diamond represent the pairwise correlation (D’ and r^2^) between 2 variants of *IRF6* in chromosome 1 and 2 SNPs of *GREM1* in chromosome 15.

We observed two pathogenic variants in *GREM1*′s exon and the *GREM1–FMN1* intergenic region: rs2280738 and rs1258763. However, neither of these variants showed an association with the Vietnamese cleft sample in this study (Table 3). Additionally, these two SNPs exhibited a moderate LD with each other, with D’ ≥ 0.59 among NSOFC phenotypes (Figure 1).

The LD plot in Figure 1 indicates that the alleles at rs2235375 and rs846810 are strongly correlated. The D′ values (≥0.88) for the pairwise LD comparisons between alleles at these SNPs are close to 1.0 (for strong LD). Because they are in strong LD, Haploview groups these SNPs into a common haplotype block. We analyzed the haplotypes based on these two SNPs of *IRF6* and two SNPs of *GREM1* to confirm the two-marker haplotypes associated with the risk of NSOFC phenotypes (Table 4). Statistical significance was mainly observed for haplotypes comprising the minor allele (G) of rs2235375 and the G allele of rs846810. The G-G (rs2235375–rs846810 of *IRF6*) haplotype was associated with the etiology of NSCL/P and NSOFC (*p*-values of 0.0003 and 0.0012, respectively). Moreover, the C-A (rs2235375–rs846810 of *IRF6*) haplotype was significant with NSCL/P (*p* = 0.0011) in the case–control analysis. For the *GREM1* gene, there was no statistical association detected with the NSOFC groups in the two-marker haplotypes (C-C, G-C and C-T) for rs2280738 and rs1258763.

### 3.4. Family-Based Association Study and Haplotype Analysis

Table 5 summarizes the results of the TDT analysis of the family-based association of *IRF6*, *TP63* and *GREM1* with NSOFC phenotypes in trios in the Vietnamese cohort. Among all five SNPs, only rs2235375 showed a significant association between NSCL/P and the G allele as the over-transmission allele, with a *p*-value of 0.002 and OR of 1.34 with 95% CI 1.11–1.63 after Bonferroni correction.

Parent-of-origin effects were also examined by separating paternal and maternal alleles. Despite being confirmed by TDT analysis, rs2235375 showed no evidence of excess parental transmission in the POO analysis (Table 5).

When conducting haplotype-based transmission disequilibrium analyses with Haploview, we failed to identify the parental over-transmission of haplotypes in *IRF6* and *GREM1* after *p*-value adjustment (Table 4).

### 3.5. SNP × SNP Epistasis

We performed pair-wise SNP × SNP epitasis between different genes to analyze numerous suggested potential interactions. However, none of the pairwise SNP × SNP interactions reached the adjusted significance (Table 6).

## 4. Discussion

An understanding of the implication of risk factors for NSOFC in diverse racial populations seems to provide more insights into the fundamental etiology. GWAS have successfully identified several related genes and loci that contribute to the high genetic heterogeneity underlying this malformation. However, it is limited in generalizability to other populations and tends to ignore susceptible genes/loci for specific NSOFC subtypes. Further studies with case–control and case–parent trio designs should be useful to unveil the causative factors of NSOFC, and it is vital to explore the differences in diverse populations regarding the same genes/markers. Besides this, linkage studies have provided new tools to detect various possible loci that could have a causal role in cleft lip and palate pathogenesis. The case–control design is the most popular approach to gene mapping in complex traits for a specific population. However, it has been shown that the weakness of this design may generate false associations as an effect of population stratification. Family-based trio design is an adequate method to address the effect of population stratification. This approach allows us to detect preferentially transmitted alleles based on parental and proband genotypes [28]. In genetic association studies, a statistically significant association is observed if the *p*-value falls below a preset threshold to reject the null hypothesis of a genetic association. Analyzing a substantial number of SNP markers results in numerous comparisons, thereby increasing the false positive rate. To avoid this issue, Bonferroni adjustment was applied to control the occurrence of false positives (type I errors). At the significance level of *p* < 0.002, the power of the association study of the significant markers in *IRF6* was more than 80%, which was calculated using the Genetic Association Study (GAS) Power Calculator from the CaTS Power Calculator [29]. Additionally, determining an appropriate sample size is crucial in the initial phase of designing a genetic association study to ensure sufficient statistical power [30].

Genetic research on oral clefts in the Vietnamese population has been limited. However, there have been some notable studies and reports that have shed light on potential genetic factors contributing to NSOFC, including the transmission distortion for alleles of *MSX1* [31]; the V274I polymorphism (rs2235371) in *IRF6* [9]; rs9429829 of *SYT14*, 17 SNPs in the 10q25.3 region and the rs227731 variant from 17q22 [32]; rs2237493 of *MEOX2* [33]; and rs1675414 of *TFAP2A* in NSCLO, as previously reported [20].

Variants in *IRF6* are associated with multiple phenotypes of OFC. For example, structural mutations of *IRF6* cause VWS or Popliteal Pterygium Syndrome. With the incomplete penetrance of VWS, the isolated OFC, lip pits alone, dental anomalies or even non-discernible phenotypes could be included in the least severe end of the VWS spectrum. Because of this variation, whether *IRF6*, one of nine members of a family of transcription factors (IRFs), causes NSOFC remains controversial. Nevertheless, many studies have shown common alleles in *IRF6* associated with NSOFC and this has made *IRF6* the most frequently studied gene related to NSOFC. This has been independently confirmed in GWAS [6] and candidate gene studies [9], while animal models have revealed that *Irf6* is involved in craniofacial morphogenesis and ectodermal formation [34]. Among these proven variants, the rs2235371 (V274I) polymorphism emerges as the most plausible marker for NSOFC. However, as Park et al. pointed out, “Significant results observed from SNPs other than rs2235371 (p.V274I) suggest that rs2235371 itself is not causal, but rather in LD with some causal mutation in *IRF6*” [35]. rs2235375, located in intron 6 of *IRF6*, has been reported to have significant LD with the V274I locus [26]. In addition, rs2235375 has shown evidence of an association, with an increase in DNA methylation and a decrease in expression in cerebellar tissues [36]. In our current study, with a Vietnamese case–control sample, rs2235375 showed a significant association with the NSCLP and NSCL/P groups, with p_allele_ values of 0.0018 and 0.0003, respectively, indicating an increase in the G allele as a risk for NSCLP and NSCL/P. More specifically, with a p_Geno_ of 0.0014, an association with a *p*-value of 0.0003 with an OR value of 1.94 (95% CI 1.35–2.80) (not shown in the table) was seen with the increase in homozygous genotype GG compared with CC in the NSCL/P phenotype. In the recessive models, NSCL/P was associated with a protective effect of rs2235375 by decreasing the number of GG genotypes (p_rec_ = 0.0011). On the one hand, these results are consistent with various previous reports in different populations, such as Italian [37], Chinese [38], Chilean [39] and Norwegian [26]. On the other hand, rs2235375 remained non-associated with the Mexican cleft population in the Velázquez-Aragón et al. study [40]. This difference could be attributed to the diversity of the racial populations. The TDT results in the triad analysis are worth mentioning, with the significant over-transmission of the G allele with NSCL/P (*p* = 0.0020; OR = 1.34), suggesting that offspring who inherited the G allele at rs2235375 had a 1.34-fold increased risk of NSCL/P compared to the C allele holders. The same results were seen in Mexican [41], European–American [37], Taiwanese, Singaporean, Korean and Western Chinese case–parent triads in a genome-wide TDT analysis [35]. However, we did not detect significant distortion in the allele transmission of rs2235375 from parents to affected progeny in any cleft phenotypes using the POO analysis. These results arise from the different approaches between the two family-based methods. TDT avoids false positive associations by testing the difference between the frequency of marker alleles transmitted from heterozygous parents to the affected children and the frequency of marker alleles not transmitted. In comparison, POO looks separately for further possible roles between paternal and maternal genetic effects.

The most noteworthy aspect of this study regarding *IRF6* is the novel SNP, which has never been reported in both previous GWAS and replicate studies, namely rs846810. rs846810 showed a strong association with the NSCL/P group (*p* = 0.0015). An increase in the frequency of the G allele at this site could be associated with an elevated risk of NSCL/P. rs846810, located in intron 5 of *IRF6*, was in LD with rs2235375 (D’ ≥ 0.88, r^2^ ≥ 0.26) throughout cleft groups. In the haplotype analysis, the increase in G-G and/or decrease in C-A haplotypes in *IRF6* contributed to the etiology of NSCL/P. Although *IRF6* rs846810 was located on 1q32.2 and had no direct influence on the structure of the protein, we used HaploReg to generate a motif analysis. HaploReg is a tool for exploring the non-coding variants and their haplotype blocks, and the effects of SNPs on regulatory motifs. HaploReg indicated that rs846810 could change the transcription factor binding site (Foxp1, rf_disc3, Irf_known9, etc.). Thus, this marker might play indirect roles in the pathogenesis of NSOFC by modulating the corresponding transcription factors of *IRF6*.

In both the case–parent and haplotype analyses of NSOFC triads, no significant association was detected for rs846810 and *IRF6* haplotypes. However, the G-G haplotype of *IRF6* was nominally significant with NSCL/P (*p* = 0.0046) and is worthy of consideration for another independent replication study in the future. This is the first time that these haplotypes in *IRF6* have been reported and rs846810 has been addressed as a causal genetic variant of NSCL/P. Therefore, rs846810 should be considered for investigation in various communities for the comparison of racial genetic diversity.

Variants in *TP63* have been reported as an etiology for variable features, including, but not limited to, ectrodactyly-ectodermal dysplasia cleft syndrome, Rapp–Hodgkin syndrome, split-hand/foot malformation, limb-mammary syndrome and ADULT syndrome [42]. *TP63* gene transcription can start from two distinct promoters, resulting in two isoforms: the TAp63 isoform and the ΔNp63 isoform. The *TP63* gene also contains versatile regulatory elements in its large intronic regions. The linkage between *IRF6* and *TP63* has been proven in numerous previous studies, especially the ΔNp63 isoform. *IRF6* was discovered to promote ΔNp63 protein degradation in both mouse palatal shelves and human keratinocytes, expressed in all embryonic stages during epidermal, tooth and hair development. In contrast, the TAp63 isoforms are not detected until the late embryonic stage [43]. The distinct contribution between the two isoforms to cleft etiopathogenesis was partly confirmed by various SNPs in the intron 1 region of *TP63*, which was proven to have no significant association with NSCLP [14,27]. rs9332461 was chosen from the upstream region of *TP63*, which contains the promoter region and various regulatory elements of *TP63.* HaploReg suggests that *TP63* rs9332461 could change the transcription factor Nkx2_1 in brain, lung and thyroid development [44]. Moreover, research in multiplex populations has shown that rs9332461 may confer an increased risk for NSCLP [27]. However, it is worth noting that the significance and association of rs9332461 can vary among different ethnicities. Therefore, additional studies in diverse populations are required to validate and expand our understanding of the role of this SNP in cleft diseases.

*GREM1*, a downstream target of BMP signaling, is required for limb bud development, where it validates a positive SHH–FGF feedback loop through the restriction of BMP signaling. *GREM1* also induces BMP-independent phenotypes, including the induction of proliferation and apoptosis [45] and the control of monocyte migration, adhesion and apoptosis via the inhibition of macrophage migration inhibitory factor release [46]. Additionally, the contribution of *GREM1* is indisputable for the embryonic process (kidney, lung and bone development). Ludwig et al. demonstrated the *GREM1* expression in the fusion region of the maxillary and medial nasal processes during mouse embryogenesis at E12.5 (embryonic day 12.5), and between E12.5 and E15.5 [47]. For the 15q13 locus, which contained rs2280738 and rs1258763 in our study, evidence for long-distance regulatory effects on *Grem1* has been provided by studies in other mammals, and the *Fmn1* gene was found necessary for the cis-regulation of *Grem1* transcription [48].

rs2280738 was chosen from the exonic region and rs1258763 was represented in the *GREM1–FMN1* intergenic region that could be deputized for *GREM1*. Neither rs2280738 nor rs1258763 showed significant differences in the distribution of alleles, genotypes, haplotype association and TDT and POO analysis among NSOFC phenotypes with our correction for multiple comparisons. Few genetic studies have been carried out regarding the impact of *GREM1* variants on the risk of NSOFC, and the results vary both in GWAS [5,47] and among different populations through association studies [32,49,50]. rs2280738 was presented as a rare *GREM1* mutation for the pedigrees of families in Ludwig et al.’s report [47] and this was replicated with the Chinese population [51]. In contrast to our results, Han Chinese citizens were positively associated with rs2280738 in the NSCLO group [51]. Our findings support the argument that *GREM1* has a low number of etiologic exonic variants in cleft patients [49,50]. Regarding rs1258763, various previous reports hold opposite opinions on this variant’s contribution to NSOFC in the European, Chinese and Brazilian populations. Ludwig et al. suggested that rs1258763 was significantly associated with an increased risk for NSCLP [47], while Mostowska et al. asserted that the minor allele (G) of rs1258763 was supported in decreasing the risk of NSOFC among the Polish [50]. Many subsequent studies have agreed with the relation between rs1258763 and cleft etiology in the Chinese [52] and Brazilian populations [53]. Furthermore, the rs1258763 minor allele is significantly associated with nasal width reduction and this association was found to be stronger in males [54]. Our negative association results suggest that its conflicting effects may result from genetic heterogeneity in different ethnicities, even in the Asian race, where the frequency of susceptibility variants may differ across populations. Of course, there are other possibilities, such as the fact that the differences may be due to the low statistical power, including our limited sample sizes and differences in the experimental design and methods. Besides these, our results indicate that the *GREM1–FMN1* intergenic region might not contain causal variants, at least in the Vietnamese population. The question of whether the 15q13.3 locus, where rs1258763 is located, plays a role in the genetic susceptibility to NSOFC remains open and requires further research.

In the present work, we also found moderate LD between *GREM1* rs2280738 and rs1258763, with 0.54 ≤ D’ ≤ 0.63 but a very low r^2^. The low r^2^ was derived from the rarer allele frequency of rs1258763 compared with rs2280738 in the Vietnamese population.

Haplotype-based association studies offer advantages over single-marker analysis by providing increased statistical power and the ability to detect rare variants that might not be well captured by single-marker analyses due to low allele frequencies, elucidating biological relevance [55], identifying interactions between SNPs at a locus, reducing the multiple testing burden and accounting for population structures more effectively [56]. However, they may introduce complexity and require larger sample sizes. The choice between the two methods depends on the research question and the genetic architecture of the trait. However, combining both approaches can yield a more comprehensive understanding of genetic associations.

OFC is a complex congenital anomaly exhibiting an interaction between genetic and environmental factors [57]. Although cleft lip and cleft palate often occur together, NSCL/P and NSCPO are two distinct groups of NSOFC based on embryological and epidemiological distinction [58]. Several GWAS and meta-analyses have shown that while common variants strongly contribute to NSCL/P, they do not seem to affect NSCPO [6,59]. NSCPO might be more often caused by rare deleterious variants and more vulnerable to environmental factors. Many studies also suggest that NSCLO and NSCLP might have separate genetic pathways [60]. The *IRF6* susceptibility in our study with NSCL/P and its subtypes (NSCLO and NSCLP) also confirmed the different genetic etiologies between NSCL/P and NSCPO.

Several limitations of our study need to be pointed out, such as the small size of the sample groups, the fact that it was not a multiracial study, the insufficient numbers of selected variant loci and the lack of analysis of the biological functions of the polymorphisms and gene–environmental factors. However, our study will open promising future research in Vietnamese cleft disorders, particularly regarding *IRF6* and its related genes. This includes exploring the *PBx–WNT–TP63–IRF6* pathway, studying the effect of *IRF6* on various genes and investigating interactions within the *SHH–GREM1–BMP4–FGF* network.

## 5. Conclusions

In conclusion, we performed case–control and family-based evaluations combined with haplotype analysis on 527 Vietnamese trios among NSOFC phenotypes. This study investigated the role of *IRF6*, *TP63* and *GREM1* variants in the etiology of NSOFC and its phenotypes. Our study provided additional evidence for the association between *IRF6* rs2235375 with NSCLP and NSCL/P and showed the significant over-transmission of the G allele with NSCL/P. We also identified the novel locus of *IRF6* (rs846810) in association with NSCL/P and the rs2235375–rs846810 haplotypes (G-G and C-A) associated with NSCL/P and NSOFC. Our data did not support the direct involvement of *TP63* and *GREM1* in orofacial cleft diseases in the Vietnamese population.

## Figures and Tables

**Table 1:** Characteristics of the study sample.

Type of Cleft	N (%)	Sex		Cleft Side	
		Male	Female	Right Side	Left Side
NSOFC	527 (100.0)	313 (59.4)	214 (40.6)	NA	NA
NSCPO	101 (19.2)	58 (57.4)	43 (42.6)	NA	NA
NSCL/P	426 (80.8)	255 (58.9)	171 (41.1)	113 (26.5)	313 (65.2)
NSCLO	172 (32.6)	100 (58.1)	72 (41.9)	46 (26.7)	126 (73.3)
NSCLP	254 (48.2)	155 (61.0)	99 (39.0)	65 (25.6)	189 (74.4)
NSUCL/P	325 (76.3)	184 (56.6)	141 (43.4)	113 (34.8)	212 (65.2)
NSBCL/P	101 (23.7)	71 (70.3)	30 (29.7)	NA	NA

Abbreviations: NA, not available; NSUCL/P, non-syndromic unilateral cleft lip with/without cleft palate; NSBCL/P, non-syndromic bilateral cleft lip with/without cleft palate.

**Table 2:** Minor allele frequency for each SNP and Hardy–Weinberg equilibrium test.

Gene	SNP	Allele	HWEp	Minor Allele Frequency					
				Control	NSOFC	NSCPO	NSCLO	NSCLP	NSCL/P
*IRF6*	rs2235375	C>G	0.7908	0.4374	0.4972	0.4010	0.5174	0.5217	0.5200
*IRF6*	rs846810	A>G	0.7906	0.2068	0.2571	0.2079	0.2762	0.2638	0.2688
*TP63*	rs9332461	G>A	0.2273	0.2362	0.2410	0.2426	0.2238	0.2520	0.2406
*GREM1*	rs2280738	C>G	0.1106	0.2315	0.2324	0.2129	0.2006	0.2618	0.2371
*GREM1*	rs1258763	C>T	0.1772	0.0721	0.0778	0.0693	0.0930	0.0709	0.0798

Abbreviations: HWEp, Hardy–Weinberg equilibrium *p*-value.

**Table 3:** Associations of *IRF6–TP63, GREM1* single-nucleotide polymorphisms (SNP) in the case–control study.

Gene		Case *(Control)*	P_Geno_ Value	OR_allele_ (95% CI) P_allele_ Value	OR_Dom_ (95% CI) P_Dom_ Value	OR_Rec_ (95% CI) P_Rec_ Value	OR_Over_ (95% CI) P_Over_ Value
NSOFC							
*IRF6*							
rs2235375	CC/CG/GG	139/252/136 *(165/263/99)*	0.0159	1.27 (1.07–1.51) 0.0060	0.79 (0.60–1.02) 0.0771	1.50 (1.12–2.02) 0.0062	0.92 (0.72–1.17) 0.4979
rs846810	AA/AG/GG	298/187/42 *(330/176/21)*	0.0113	1.33 (1.08–1.63) 0.0062	0.78 (0.60–0.99) 0.0446	2.09 (1.21–3.58) 0.0064	1.10 (0.85–1.41) 0.4758
*TP63*							
rs9332461	GG/AG/AA	297/206/24 *(302/201/24)*	0.9497	1.03 (0.84–1.25) 0.7983	0.96 (0.75–1.23) 0.7558	1.00 (0.56–1.78) 1	1.04 (0.81–1.33) 0.7518
*GREM1*							
rs2280738	CC/CG/GG	309/191/27 *(318/174/35)*	0.3766	1.01 (0.82–1.23) 0.9588	0.93 (0.73–1.19) 0.5723	0.76 (0.45–1.27) 0.295	1.15 (0.89–1.49) 0.2711
rs1258763	CC/CT/TT	450/72/5 *(456/66/5)*	0.8604	1.09 (0.78–1.50) 0.6191	0.90 (0.64–1.29) 0.5948	1.00 (0.29–3.47) 1	1.11 (0.77–1.58) 0.5838
NSCPO							
*IRF6*							
rs2235375	CC/CG/GG	38/45/18 *(165/263/99)*	0.4533	0.86 (0.63–1.17) 0.3388	1.32 (0.85–2.06) 0.2139	0.94 (0.54–1.63) 0.8197	0.81 (0.53–1.24) 0.3244
rs846810	AA/AG/GG	64/32/5 *(330/176/21)*	0.8722	1.01 (0.69–1.46) 0.9721	1.03 (0.66–1.60) 0.8868	1.25 (0.46–3.41) 0.6554	0.92 (0.59–1.46) 0.7375
*TP63*							
rs9332461	GG/AG/AA	56/41/4 *(302/201/24)*	NA	1.04 (0.73–1.47) 0.8464	NA	NA	1.11 (0.72–1.71) 0.6425
*GREM1*							
rs2280738	CC/CG/GG	62/35/4 *(318/174/35)*	NA	0.90 (0.62–1.29) 0.5635	NA	NA	1.08 (0.69–1.68) 0.7492
rs1258763	CC/CT/TT	88/12/1 *(456/66/5)*	NA	0.96 (0.53–1.73) 0.8876	NA	NA	0.94 (0.49–1.81) 0.8577
NSCLO							
*IRF6*							
rs2235375	CC/CG/GG	40/86/46 *(165/263/99)*	0.0329	1.38 (1.08–1.76) 0.0096	0.66 (0.45–0.99) 0.0440	1.58 (1.06–2.40) 0.0254	1.00 (0.71–1.41) 0.9828
rs846810	AA/AG/GG	93/63/16 *(330/176/21)*	0.0117	1.46 (1.11–1.93) 0.0074	0.70 (0.50–0.99) 0.0464	2.47 (1.26–4.85) 0.0068	1.15 (0.80–1.65) 0.4379
*TP63*							
rs9332461	GG/AG/AA	101/65/6 *(302/201/24)*	0.8219	0.93 (0.70–1.25) 0.6366	1.06 (0.75–1.50) 0.7443	0.76 (0.30–1.89) 0.5493	0.99 (0.69–1.40) 0.9346
*GREM1*							
rs2280738	CC/CG/GG	109/57/6 *(318/174/35)*	0.3022	0.83 (0.62–1.13) 0.2323	1.14 (0.80–1.62) 0.4791	0.51 (0.21–1.23) 0.1265	1.01 (0.70–1.45) 0.9763
rs1258763	CC/CT/TT	142/28/2 *(456/66/5)*	NA	1.32 (0.86–2.03) 0.2071	NA	NA	1.35 (0.84–2.19) 0.2100
NSCLP							
*IRF6*							
rs2235375	CC/CG/GG	61/121/72 *(165/263/99)*	0.0053	1.40 (1.13–1.74) **0.0018**	0.69 (0.49–0.98) 0.0352	1.71 (1.21–2.43) 0.0025	0.91 (0.68–1.23) 0.5527
rs846810	AA/AG/GG	141/92/21 *(330/176/21)*	0.0215	1.37 (1.07–1.76) 0.0116	0.75 (0.55–1.00) 0.0572	2.17 (1.16–4.06) 0.0129	1.13 (0.83–1.55) 0.4362
*TP63*							
rs9332461	GG/AG/AA	140/100/14 *(302/201/24)*	0.7652	1.09 (0.85–1.39) 0.4962	0.92 (0.68–1.24) 0.5634	1.22 (0.62–2.41) 0.5600	1.05 (0.77–1.43) 0.7408
*GREM1*							
rs2280738	CC/CG/GG	138/99/17 *(318/174/35)*	0.2463	1.18 (0.92–1.50) 0.1897	0.78 (0.58–1.06) 0.1104	1.01 (0.55–1.84) 0.9784	1.30 (0.95–1.77) 0.1018
rs1258763	CC/CT/TT	220/32/2 *(456/66/5)*	NA	0.98 (0.65–1.48) 0.9291	NA	NA	1.01 (0.64–1.58) 0.9764
NSCL/P							
*IRF6*							
rs2235375	CC/CG/GG	101/207/118 *(165/263/99)*	**0.0014**	1.39 (1.16–1.67) **0.0003**	0.68 (0.51–0.91) 0.0093	1.66 (1.22–2.25) **0.0011**	0.95 (0.73–1.22) 0.6868
rs846810	AA/AG/GG	234/155/37 *(330/176/21)*	0.0031	1.41 (1.14–1.74) **0.0015**	0.73 (0.56–0.94) 0.0164	2.29 (1.32–3.98) 0.0025	1.14 (0.87–1.49) 0.3354
*TP63*							
rs9332461	GG/AG/AA	241/165/20 *(302/201/24)*	0.9735	1.02 (0.83–1.27) 0.8239	0.97 (0.75–1.25) 0.8203	1.03 (0.56–1.90) 0.9180	1.03 (0.79–1.33) 0.8518
*GREM1*							
rs2280738	CC/CG/GG	247/156/23 *(318/174/35)*	0.427	1.03 (0.83–1.28) 0.7744	0.90 (0.70–1.18) 0.4609	0.80 (0.47–1.38) 0.4251	1.17 (0.90–1.53) 0.2452
rs1258763	CC/CT/TT	362/60/4 *(456/66/5)*	NA	1.12 (0.79–1.57) 0.5268	NA	NA	1.15 (0.79–1.67) 0.4794

Abbreviations: NA, not available; Geno, genotypic; Dom, dominant; Rec, recessive; Ove, over-dominant. In bold are *p*-values that were significant after adjustment with Bonferroni correction in multiple tests (*p* ≤ 0.002).

**Table 4:** The haplotype association analysis in case–control and case–parent samples.

Gene/Haplotype		Haplotype Freq.	Case, Control Freq.	P_CC_ Value	T/U	P_TDT_ Value
NSOFC						
*IRF6*	rs2235375–rs846810					
	C-A	0.522	0.497, 0.548	0.0183	240.0/281.7	0.0679
	G-A	0.246	0.246, 0.245	0.9553	191.2/177.5	0.4753
	G-G	0.221	0.251, 0.192	**0.0012**	198.5/164.6	0.0747
*GREM1*	rs2280738–rs1258763					
	C-C	0.699	0.695, 0.703	0.6844	217.5/206.8	0.6026
	G-C	0.226	0.227, 0.224	0.8941	185.7/169.4	0.3855
	C-T	0.069	0.072, 0.065	0.5151	63.4/85.3	0.0725
NSCPO						
*IRF6*	rs2235375–rs846810					
	C-A	0.555	0.596, 0.547	0.2017	59.2/42.9	0.1077
	G-A	0.238	0.196, 0.246	0.1277	29.2/31.4	0.7719
	G-G	0.193	0.205, 0.191	0.6580	31.0/45.0	0.1083
*GREM1*	rs2280738–rs1258763					
	C-C	0.707	0.724, 0.704	0.5566	37.9/34.3	0.6635
	G-C	0.221	0.206, 0.224	0.5777	32.4/27.1	0.4963
	C-T	0.064	0.063, 0.065	0.9189	10.2/18.0	0.1410
NSCLO						
*IRF6*	rs2235375–rs846810					
	C-A	0.529	0.473, 0.547	0.0171	70.0/104.9	0.0082
	G-A	0.247	0.251, 0.246	0.8635	67.6/61.1	0.5671
	G-G	0.210	0.267, 0.191	0.0028	74.4/46.9	0.0125
*GREM1*	rs2280738–rs1258763					
	C-C	0.705	0.711, 0.704	0.7839	73.1/64.1	0.4433
	G-C	0.217	0.196, 0.224	0.2627	54.0/54.9	0.9327
	C-T	0.071	0.088, 0.065	0.1455	26.1/33.1	0.3642
NSCLP						
*IRF6*	rs2235375–rs846810					
	C-A	0.523	0.471, 0.547	0.0049	110.8/133.9	0.1399
	G-A	0.252	0.265, 0.246	0.4189	94.4/84.9	0.4749
	G-G	0.213	0.257, 0.191	0.0031	93.1/72.6	0.1124
*GREM1*	rs2280738–rs1258763					
	C-C	0.694	0.674, 0.704	0.2278	106.5/108.4	0.8983
	G-C	0.234	0.256, 0.224	0.1718	99.4/87.4	0.3822
	C-T	0.065	0.065, 0.065	0.9826	27.1/34.2	0.3627
NSCL/P						
*IRF6*	rs2235375–rs846810					
	C-A	0.514	0.472, 0.548	**0.0011**	180.8/238.8	0.0046
	G-A	0.251	0.259, 0.246	0.5112	162.0/146.0	0.3612
	G-G	0.223	0.261, 0.192	**0.0003**	167.5/119.5	0.0046
*GREM1*	rs2280738–rs1258763					
	C-C	0.697	0.689, 0.703	0.4861	179.6/172.4	0.7008
	G-C	0.228	0.231, 0.224	0.7154	153.3/142.4	0.5239
	C-T	0.069	0.074, 0.065	0.4333	53.2/67.4	0.1958

Abbreviations: CC, case–control; TDT, transmission disequilibrium test; Freq., frequency; T/U, transmitted/not transmitted. In bold are *p*-values that were significant after adjustment with Bonferroni correction in multiple tests (*p* ≤ 0.0017).

**Table 5:** The transmission disequilibrium test (TDT) and the parent-of-origin (POO) likelihood ratio test of inequality between paternal and maternal.

Gene	SNP	A1/A2	TDT			POO				
						Paternal		Maternal		P_POO_
			T/U	OR (95% CI)	P_TDT_	T/U	P_Pat_	T/U	P_Mat_	
NSOFC										
*IRF6*	rs2235375	G/C	290/243	1.19 (1.01–1.42)	0.0418	138.5/120.5	0.2634	151.5/122.5	0.0798	0.6738
	rs846810	G/A	201/173	1.16 (0.95–1.42)	0.1477	94/89	0.7117	107/84	0.0961	0.3670
*TP63*	rs9332461	A/G	197/197	1.00 (0.82–1.22)	1.0000	100/98	0.8870	97/99	0.8864	0.8403
*GREM1*	rs2280738	G/C	185/174	1.06 (0.86–1.31)	0.5615	95/87	0.5532	90/87	0.8216	0.7980
	rs1258763	T/C	66/93	0.71 (0.52–0.97)	0.0323	33/56	0.0148	33/37	0.6326	0.2020
NSCPO										
*IRF6*	rs2235375	G/C	42/59	0.71 (0.48–1.06)	0.0907	16/29	0.0526	26/30	0.5930	0.2717
	rs846810	G/A	31/45	0.69 (0.44–1.09)	0.1083	12.5/24.5	0.0485	18.5/20.5	0.7488	0.2279
*TP63*	rs9332461	A/G	39/39	1.00 (0.64–1.56)	1.0000	17/21	0.5164	22/18	0.5271	0.3657
*GREM1*	rs2280738	G/C	30/26	1.15 (0.68–1.95)	0.5930	14.5/12.5	0.7003	15.5/13.5	0.7103	0.9847
	rs1258763	T/C	11/20	0.55 (0.26–1.15)	0.1060	6/11	0.2253	5/9	0.2850	0.9806
NSCLO										
*IRF6*	rs2235375	G/C	106/72	1.47 (1.09–1.99)	0.0108	55.5/33.5	0.0197	50.5/38.5	0.2034	0.4454
	rs846810	G/A	76/48	1.58 (1.10–2.27)	0.0119	40.5/22.5	0.0233	35.5/25.5	0.2004	0.4868
*TP63*	rs9332461	A/G	63/59	1.07 (0.75–1.52)	0.7172	34/31	0.7098	29/28	0.8946	0.8747
*GREM1*	rs2280738	G/C	54/56	0.96 (0.66–1.40)	0.8488	30.5/30.5	1.0000	23.5/25.5	0.7751	0.8315
	rs1258763	T/C	26/34	0.76 (0.46–1.27)	0.3017	12.5/21.5	0.1227	13.5/12.5	0.8445	0.2424
NSCLP										
*IRF6*	rs2235375	G/C	142/112	1.27 (0.99–1.62)	0.0598	67/58	0.4208	75/54	0.0645	0.4665
	rs846810	G/A	94/80	1.18 (0.87–1.58)	0.2885	41/42	0.9126	53/38	0.1159	0.2430
*TP63*	rs9332461	A/G	95/99	0.96 (0.72–1.27)	0.7740	49/46	0.7582	46/53	0.4817	0.4764
*GREM1*	rs2280738	G/C	101/92	1.10 (0.83–1.46)	0.5171	50/44	0.5360	51/48	0.7630	0.8157
	rs1258763	T/C	29/39	0.74 (0.46–1.20)	0.2253	14.5/23.5	0.1443	14.5/15.5	0.8551	0.4005
NSCL/P										
*IRF6*	rs2235375	G/C	248/184	1.34 (1.11–1.63)	**0.0020**	122.5/91.5	0.0341	125.5/92.5	0.0254	0.9454
	rs846810	G/A	170/128	1.33 (1.06–1.67)	0.0150	81.5/64.5	0.1594	88.5/63.5	0.0426	0.6755
*TP63*	rs9332461	A/G	158/158	1.00 (0.80–1.25)	1.0000	83/77	0.6353	75/81	0.6310	0.4997
*GREM1*	rs2280738	G/C	155/148	1.05 (0.84–1.31)	0.6876	80.5/74.5	0.6299	74.5/73.5	0.9345	0.7809
	rs1258763	T/C	55/73	0.75 (0.53–1.07)	0.1116	27/45	0.0339	28/28	1.0000	0.1576

Abbreviations: TDT, transmission disequilibrium test; POO, parent of origin; Pat, paternal; Mat, maternal. A1/A2, 2 alleles for each SNP; T/U, transmitted/not transmitted. In bold are *p*-values that were significant after adjustment with Bonferroni correction in multiple tests (*p* ≤ 0.002).

**Table 6:** The SNP × SNP epitasis for case–control population-based sample.

Gene 1	SNP1	Gene 2	SNP2	P_NSOFC_	P_NSCPO_	P_NSCLO_	P_NSCLP_	P_NSCL/P_
*IRF6*	rs2235375	*TP63*	rs9332461	0.1871	0.9353	0.3621	0.1295	0.1200
	rs846810		rs9332461	0.6215	0.7205	0.6412	0.5306	0.4804
	rs2235375	*GREM1*	rs2280738	0.7350	0.3522	0.2438	0.4572	0.9533
	rs2235375		rs1258763	0.6445	0.6755	0.5768	0.9947	0.7338
	rs846810		rs2280738	0.5479	0.3734	0.4334	0.3005	0.7436
	rs846810		rs1258763	0.1696	0.0851	0.1996	0.5862	0.2867
*TP63*	rs9332461	*GREM1*	rs2280738	0.7330	0.4430	0.3672	0.6451	0.4836
	rs9332461		rs1258763	0.7343	0.9310	0.6662	0.8212	0.7264

Values of *p* ≤ 0.002 are significant after Bonferroni correction for multiple testing.

## Data Availability

The data that support the findings of this study are available from the corresponding author upon reasonable request.
